# Cyberknife Stereotactic Body Radiation Therapy for Nonresectable Tumors of the Liver: Preliminary Results

**DOI:** 10.1155/2010/309780

**Published:** 2010-06-28

**Authors:** K. Goyal, D. Einstein, M. Yao, C. Kunos, F. Barton, D. Singh, C. Siegel, J. Stulberg, J. Sanabria

**Affiliations:** ^1^Departments of Surgery, University Hospitals-Case Medical Center, Case Western Reserve University, Cleveland, OH 44106, USA; ^2^Departments of Radiation Oncology, University Hospitals-Case Medical Center, Case Western Reserve University, Cleveland, OH 44106, USA; ^3^Departments of Medicine, University Hospitals-Case Medical Center, Case Western Reserve University, Cleveland, OH 44106, USA

## Abstract

*Purpose*. Stereotactic body radiation therapy (SBRT) has emerged as a treatment option for local tumor control of primary and secondary malignancies of the liver. We report on our updated experience with SBRT in patients with non-resectable tumors of the liver. *Methods*. Our first 17 consecutive patients (mean age 58.1 years) receiving SBRT for HCC (*n* = 6), IHC (*n* = 3), and LM (*n* = 8) are presented. Mean radiation dose was 34Gy delivered over 1–3 fractions. *Results*. Treated patients had a mean decrease in maximum pretreatment tumor diameter from 6.9 ± 4.6 cm to 5.0 ± 2.1 cm at three months after treatment (*P* < .05). The mean total tumor volume reduction was 44% at six months (*P* < .05). 82% of all patients (14/17) achieved local control with a median follow-up of 8 months. 100% of patients with HCC (*n* = 6) achieved local control. Patients with surgically placed fiducial markers had no complications related to marker placement. *Conclusion*. Our preliminary results showed that SBRT is a safe and effective local treatment modality in selected patients with liver malignancies with minimal adverse events. Further studies are needed to define its role in the management of these malignancies.

## 1. Introduction

An excess of 80,000 individuals are diagnosed annually with tumors of the liver in the United States [1]. Less than 20% of those neoplasms are amenable to definitive surgical management due to advanced stage of local disease or comorbid medical conditions [2]. Unresectable malignant tumors of the liver carry a poor prognosis with limited nonsurgical effective treatment options. Alternative modalities being used for treatment of unresectable liver tumors include trans-arterial chemoembolization (TACE), Y90 microspheres embolization, radiofrequency ablation (RFA), and transcutaneous ethanol injection (TEI) [3]. However, these therapies have limitations depending on size, location, number, and distribution of lesions. At this time, successful systemic therapies for unresectable primary hepatobiliary malignancies are poorly developed. Sorafenib is now routinely offered to patients with HCC based on the randomized results from the SHARP trial [4]. These forms of therapy have not had a significant impact on survival with a median overall survival of less than 1 year for nonsurgically removed primary tumors of the liver [3].

Stereotactic radiosurgery (SRS) is a technique that allows precise delivery of a large ablative radiation dose to the tumor while sparing normal surrounding tissue in 1 to 5 fractions. When used to treat brain metastases, SRS treatment results in extremely high local control rates in excess of 80%–90% [5]. Its use in extracranial tumors had been limited due to the inherent movement of abdominal organs and associated tumor movement that occurs during the respiratory cycle. One newer device that tracks tumors during respiration and automatically adjusts during patient positioning is the CyberKnife robotic radiosurgery system, which consists of three key components: (1) an advanced, lightweight linear accelerator (LINAC), (2) a robotic arm which can point the LINAC from a wide variety of angles, and (3) a tumor tracking system. The system tracks a patient's abdominal tumor during respiration using three different mechanisms: (1) An in-room kv imaging system, which is used for fiducial tracking, (2) Infrared Light Emitting Diodes (LEDs) placed on the patient's chest (synchrony vest) and wall-mounted infrared detector which allows for the construction of a patient's breathing model, and (3) a Software that creates an algorithm linking the tumor movement with the chest wall movement, so that tumor position can be predicted at all stages of the breathing cycle. With the Cyberknife system, SBRT is delivered in the setting of near real-time tracking of implanted fiducial markers combined with respiratory motion modeling to achieve submillimeter accuracy by continually detecting and correcting for tumor motion throughout treatment. It was reported that the average treatment delivery precision was 0.3 ± 0.1 mm as measured at three different SBRT facilities for spinal lesions [6]. SBRT delivered via the Cyberknife and other radiosurgery systems has been used in the treatment of several abdominal tumors including pancreatic, renal, hepatic, adrenal, and pelvic malignancies among others. We report our initial experience with 17 patients who underwent SBRT for unresectable tumors of the liver.

## 2. Materials and Methods

### 2.1. Patient Population

Medical records of the first seventeen consecutive patients treated between October 2007 and May 2009 who underwent Cyberknife SBRT for nonresectable tumors of the liver were reviewed under an IRB-approved protocol. Enrollment criteria for SBRT included (1) biopsy proven malignancy; (2) nonresectable liver disease, and (3) life expectancy of at least 12 weeks. Tumor pathology was represented by HCC (*n* = 6), IHC (*n* = 3), and secondary liver tumors (LM, *n* = 8). Patients treated for LM were found to have primary tumors from the GI tract (pancreas and colorectal, *n* = 2 and 1, resp.), from the ovary (*n* = 1), breast (*n* = 2), and lung (*n* = 2). 

Four patients underwent surgical resection for abdominal malignancy prior to SBRT. A patient with IHC underwent an R0 liver resection to be found to have multifocal disease 14 months after initial surgery and 13 months prior to SBRT. Two patients underwent pancreatico-duodenectomy for pancreatic adenocarcinoma prior to SBRT for liver metastases. One patient with liver metastases had sequential liver resection and RFA 21, 19, and 4 months prior to SBRT. 76% (13/17) of all patients underwent chemotherapy and 52% of them (9/17) underwent locoregional treatments, that is, RFA, TACE prior to SBRT.

### 2.2. Stereotactic Body Radiation Therapy (SBRT)

Patients were clinically evaluated by an HPB/transplant surgeon, a medical oncologist, hepatologist, and a radiation oncologist and staged by imaging that consisted of contrast-enhanced computerized tomography (CT scan), magnetic resonance imaging (MRI), or positron emission tomography scan (PET). Patients were then discussed at a multidisciplinary GI tumor board prior to SBRT. Subsequent imaging for contouring, treatment plan development, and implementation was obtained as needed. 

An average of 5, 3–5 mm cylindrical solid gold fiducial markers (Best Medical International, Springfield, VA) were placed either surgically (*n* = 12) or percutaneously under CT guidance (*n* = 5) within a 10 cm radius within or around the tumor tissue at a minimum distance between adjacent fiducials of 2 cm. Spinal needles and their obturator were adapted to harbor fiducials. In the OR, needles were placed 1-2 mm into the liver substance and fiducials were pushed in with the obturator. After needles were removed, hemostasis was achieved with an Argon photo coagulator. One week was provided between markers placement and SBRT treatment planning simulation to allow for fiducial settling. Patients were then brought to the SBRT suite, they were immobilized using an alpha cradle and fitted with a synchrony vest during simulation and treatment. Patients underwent imaging in the SBRT-immobilized position. These scans were imported into the Multiplan 2.05 treatment planning system and digitally fused. Tumor definition, normal tissue constraints, and final treatment plan were approved by the attending radiation oncologist, the attending hepatobiliary surgeon, and the medical physicist. 100 to 300 6 MV X-ray beams were used for each plan. Multiple fraction treatments were performed on consecutive weekdays. Prior to each treatment fraction, patients were premedicated with 4 mg of dexamethasone and 4 mg of Ondansetron. During SBRT treatment they were continuously monitored under real-time kilovoltage cameral fiducial tracking and near real-time respiratory motion modeling using a separate synchrony camera system. Average treatment time per patient and fraction was 2 hours. 

#### 2.2.1. Assessment of Response

Patients were assessed every 3 months after completion of treatment by physical exam and imaging. CT, MRI, and/or PET scans were performed at each follow-up. Total volume of the tumor was determined by Multiplan treatment planning system v2.05 (Sunnyvale, CA). The maximum tumor diameter was measured and the tumor volume was calculated by importing the image into ADAC pinnacle radiotherapy planning software with 3D volume algorithms. 

Local response to SBRT was graded by RECIST (Response Evaluation Criteria in Solid Tumors) criteria to describe change in treated tumor lesion [7]. This grading system has four tumor response grades: *Progressive disease* (at least 20% increase of the lesion), *stable disease*, *partial response* (at least 30% decrease of the target lesion), and *complete response *(disappearance of all target lesions). To further evaluate *partial tumor response* to SBRT and for the purpose of these studies, we developed a grading system from I to III and for tumor recurrence from 0 to 4 based on imaging modalities. Partial response grade I was considered a decrease in tumor volume/size <30%, but >10% from original tumor volume/size. Partial response grade II was considered as a decrease in volume/size ≥30% but <50% from original tumor volume/size. Partial response grade III was considered as a decrease in tumor volume/size ≥50%. In some instances in patients with IHC, the tumor size was similar to the original size but the enhancement and PET activity vanished; we considered these particular cases as a grade III partial response. 

Local or distal recurrent disease was graded as well. Local recurrence to treatment was defined as tumor progression within or at the periphery of the radiation field. No recurrence of the tumor was considered grade 0; local recurrence was considered grade 1 with two subgroups: grade 1a = 1 local tumor recurrence and grade 1b ≥ 1 local tumor recurrence. Distant intraabdominal recurrence was considered grade 2 and it was defined as new tumor distant (>3 cms) from the radiation field or in another organ. Furthermore, distant extra-abdominal recurrence was graded as 3. A combination of local and distant recurrence was considered grade 4.

#### 2.2.2. Adverse Events

Adverse events after SBRT were graded on a 1–5 scale according to the NCI common terminology criteria for adverse events v3.0. Causes were attributed to either surgery, placement of fiducial markers, chemotherapy, radiation induced, or related to medical comorbidities.

### 2.3. Statistical Assessment

A database of clinical, imaging, and radiation variables was created and maintained in a prospective manner. Data were exported to a main frame computer to be analyzed. Statistical routines were performed using paired *t*-test with SPSS V16.0 (Chicago, IL) and they were considered significant at a probability of <.05 level.

## 3. Results

**Figure 1:**
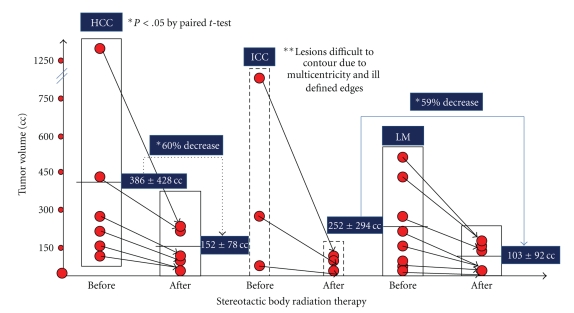
Tumor response in patients with nonresectable liver tumors treated with Stereotactic Body Radiation Therapy (SBRT). All patients with hepatocellular carcinoma (HCC) responded to SBRT with a mean decrease in tumor volume of 60% at 3 months after therapy (*P* < .05 by paired *t*-test). Similar response was observed in patients with liver metastases (LMs) treated with SBRT with a mean decrease tumor volume of 59% at 3 months posttreatment (*P* < .05 by paired *t*-test). It was difficult to be certain of the tumor response of patients with intrahepatic cholangiocarcinoma (IHC) due to the multicentricity of these tumors and the ill-defined edges.

**Figure 2:**
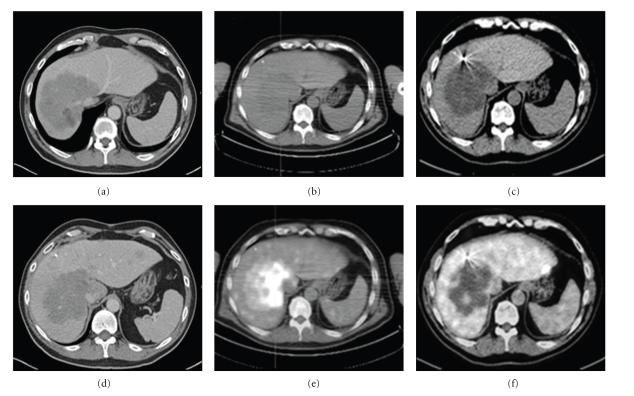
Tumor response in a patient with nonresectable and large ICC treated with Stereotactic Body Radiation Therapy (SBRT). (a) CT scan of the liver showed tumor compromising the liver outflow (right, middle, and left hepatic veins) with a satellite lesion in segment 3. (b)and (C) CT scans of the ICC before and after fiducial markers placement. (d) Tumor response by Ct scan and PET scan before (e) and at 3 months after SBRT (f). Note a “hot” liver mass before SBRT and same liver mass “cold” after treatment. It was found that the precise definition of the mass contour was difficult to establish. Although the gross total volume of the mass and its diameter appears to be similar when sizes were compared before and after treatment, its active tumor load has decreased.

Seventeen patients were treated with SBRT for nonresectable tumors of the liver. They consisted of 9 men and 8 women (*n* = 17) with a mean age of 58.1 years (range, 42 to 81 years). Treated tumors received a median prescription dose of 34 Grays (24–45 Gy) in 1 to 3 (median 3) fractions to a median prescription isodose line of 70%. Patient demographics and tumors characteristics are summarized in Table 1. Of the 6 patients treated with SRT for HCC, all of them responded to treatment with a decrease in tumor volume from 386 ± 428 cc to 152 ± 78 cc (Figure 1). Tumor volume decreased a mean of 60% three months after treatment (*P* < .05 by paired *t*-test). Similar response rates were observed in patients with liver metastases. Their tumor volume decreased from 252 ± 292 cc to 103 ± 92 cc with a mean tumor shrinkage of 59% at 3 months after SBRT (*P* < .05 by paired *t*-test). It was difficult to evaluate the response to SBRT in patients with IHC since these tumors presented in a multicentric fashion with ill-defined borders. At follow-up, tumors treated were similar in size but with no enhancement in delay films and with negative PET scans (Figure 2). In the late group of patients, tumor progression of satellite lesions was the role. 

Initial local control rate to SBRT was 82% (14/17) with a median follow-up of 8 months (range 3–20 months) (Table 2). Three patients experienced local recurrence at 1, 4, and 6 months after SBRT. Two recurrences occurred in patients with secondary tumor of the liver and one in a patient with IHC. One patient with a liver metastasis from nonsmall cell lung cancer received a second course of SBRT (24 Gy in 3 fractions) 7 months after initial SBRT for a local recurrence at the superior edge of the initially treated tumor. This patient also developed distant bone metastasis and, thus, was classified as tumor recurrence grade 4. Another patient with liver metastasis from pancreatic adenocarcinoma had local tumor recurrence at 3 months follow-up imaging. This patient had 2 tumors in two distant locations within the liver, which were treated in two separate SBRT sessions and developed distant intraabdominal metastasis and was classified as tumor recurrence grade 4. The third local failure was a patient with IHC who developed multifocal local recurrence within edges of radiosurgical field 4 months after SBRT and classified as tumor recurrence grade 1b. 

Seven patients experienced distant recurrences with mean time to distant progression of 4 months (range 1–7 months) with tumor metastases mainly to the bone (pelvic bone, clavicle, and/or ribs). Distant recurrence occurred in 33% (2/6) of patients with HCC at 6 and 7 months, respectively. One of these patients had intraabdominal distant metastasis, classified as tumor recurrence grade 2. The other patient with HCC and distant metastasis had extra-abdominal metastasis and tumor recurrence grade 3. One patient with IHC developed bone metastases 4 months after SBRT, classified as tumor recurrence grade 2. Distant recurrence occurred in 50% (4/8) of patients with secondary tumor of the liver at a mean time of 4 months. Two patients with pancreatic primary developed distant intraabdominal metastasis within 3 months of SBRT. The two other distant recurrences had primary tumors of colorectal and lung origin. All distant extra-abdominal bone metastases were treated with conventional radiotherapy. 

Regarding treatment-related toxicity, two complications were observed during percutaneous placement of fiducial markers: one fiducial migration and one bleeding complication requiring angiographic coil (Table 2). Patient admissions to the hospital following SBRT were due to medical complications and not attributable to SBRT. One patient with liver metastasis from ovarian primary developed a fluid collection after receiving an intraabdominal cycle of chemotherapy which was drained by interventional radiology. One patient was admitted for bleeding duodenal ulcer. This patient had two courses of SBRT from recurrent liver metastasis from lung primary. He responded to medical therapy for H. pylori infection. Two other patients had medical therapy for gastric and duodenal ulcers. One patient had HCC and the other had liver metastasis from colorectal primary. Three patients with baseline pain had pain management procedures performed after SBRT including celiac plexus block and intrathecal pump. Eight patients expired at a mean time of six months after SBRT. Each death was reviewed and considered definitively not related to SBRT complications.

## 4. Discussion

Preliminary analysis of 17 patients treated with SBRT for unresectable tumors of the liver is presented. All patients with HCC (*n* = 6) responded to SBRT as judged by a 60% decrease in tumor volume. Similar response rates were observed in patients with liver metastases treated with SBRT with a mean 59% reduction in their volume after SBRT. At 3 months follow-up one patient had local tumor recurrence on imaging after SBRT and two patients had distant intraabdominal recurrence. At six months follow-up three patients recurred locally and six patients recurred distantly. We noted one (6%) grade 3 adverse events due to fiducial placement, two grade 2 GI ulcers, and one grade 3 GI ulcer. On review, radiation doses to duodenal mucosa were below normal tolerance dose for that organ, but can be considered as a possible side effect of SBRT.

**Figure 3:**
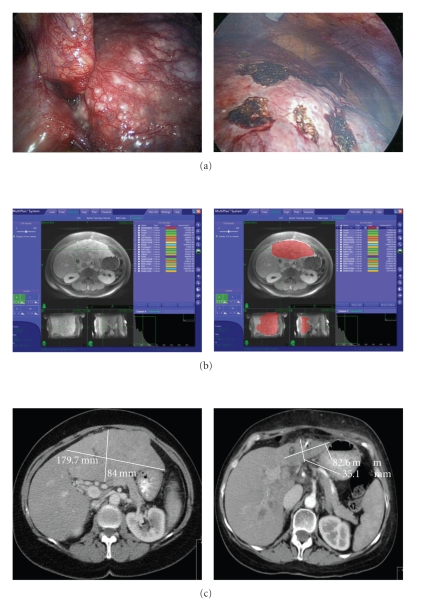
Tumor response in a patient with nonresectable and large HCC treated with Stereotactic Body Radiation Therapy (SBRT). (a) The patient underwent laparoscopy, liver biopsy, intraoperative US and fiducial markers placement. (b) The contour of liver tumor was performed and the development of the SBRT plan was approved. (c) Tumor response at 3 months after surgery. Tumor volume decreased from 1,293 cc to 258 cc.

There are only a few previous studies reporting on the use of SBRT for primary liver tumors. In 1998 Blomgren et al. reported no local failures in 11 patients with intrahepatic primary malignancies. All tumors had either growth arrest, reduction in size, or disappearance of tumor with a mean survival of 13.4 months after a mean follow-up of 12 months [8]. Romero et al. reported on 11 HCC patients with a local control rate of 75% after 22 months [9]. Tse et al. reported a series of 41 patients with HCC (*n* = 31) or IHC (*n* = 10) with a 1 year local control rate of 65% and a median survival of 11.7 and 15.0 months, respectively [10]. Two patients experienced transient biliary obstruction, which was thought to be due to radiation-induced edema. This was avoided by the use of pretreatment steroids in subsequent courses of SBRT. In our study, all six patients with HCC achieved local control after a median follow-up of 9 months after SBRT. One patient with IHC failed to respond locally. Failure to achieve local control in patients with IHC may be due to the multifocal nature of this tumor's biology. Taken together with the current literature, SBRT should be considered a viable treatment option for patients with unresectable HCC, unifocal IHC, and metastasis to liver from colorectal, breast, lung, and ovarian primaries. Based on the significant reduction of HCC tumor volume and size after SBRT in our study (Figure 3), we have began to use SBRT as another downstaging modality, in addition to TACE, for tumors over 5 cm in maximal diameter in order to make a patient eligible for liver transplantation according to Milan criteria. 

The majority of previous reports using SBRT to treat liver tumors involve the treatment of liver metastases. Reported local control rates after SBRT-treated liver metastasis range from 60%–94% at 2 years [2, 9, 11–17]. Two recent phase I/II studies reported low incidence of toxicity after SBRT with a median survival of 20.5 and 17.6 months, respectively [16, 17]. Both groups noted breast primaries had longer survival than GI (colorectal, esophageal, HCC) primaries. 75% (6/8) patients treated for liver metastasis in our study achieved local control. The local failures in our study were from lung and pancreatic adenocarcinoma primaries. Both patients who were treated with SBRT for liver metastasis after pancreaticoduodenectomy and chemoradiation (for pancreatic adenocarcinoma) developed distant intraabdominal metastasis rapidly within 3 months of SBRT. This may reflect the highly aggressive nature of the pancreatic primary.

Locoregional therapy with PEI, TACE, or RFA has the goal of eradicating tumor, while delaying distant progression by decreasing tumor burden [18]. Nine patients (33%) in our study had prior treatment with other locoregional modalities prior to SBRT: seven patients had RFA for primary or secondary malignancy of liver and two patients with HCC had TACE prior to SBRT. All nine patients developed local tumor progression with these other modalities prior to SBRT. One patient with previous RFA for lung adenocarcinoma recurred locally and distantly 6 month after SBRT. In a study by Choi et al., 20 patients were initially treated with TACE, TEI, or RFA with a local control rate of 80% after SBRT [19]. This suggests a role for SBRT as salvage therapy for local recurrence after TACE, TEI, or RFA. Due to high recurrence rates after TACE and RFA in patients with primary or secondary liver malignancies, a complementary local therapy with SBRT in tumors greater than 3 cm may be in order to decrease risk of local recurrence. The role of SBRT as primary form of therapy deserves further study.

For patients with HCC, sorafenib, a creatine-kinase inhibitor is now routinely offered based on results from the randomized results from the SHARP trial [4]. This study demonstrated an increase in survival from 10.7 months in sorafenib group compared to 7.9 months in the placebo group (*P* < .001). Nearly half of the patients with HCC consented to receive systemic therapy with sorafenib. We acknowledge that many of these patients with locally advanced cancer have other medical comorbidities or poor performance status that may preclude chemotherapy. It is our practice, all patients with unresectable hepatobiliary malignancies are offered multimodal treatment including chemotherapy when medically feasible in addition to the locally ablative treatment of SBRT. The oncology community should be aware that SBRT is a different treatment approach than conventional radiotherapy because the radiation dose in radiosurgery is ablative. Thus, using SBRT earlier in the natural history of cancer progression (before second-line chemotherapy) may lead to improved outcomes [17].

SBRT for unresectable hepatobiliary tumors achieved excellent local control rates with low adverse events from radiotherapy or fiducial marker placement [2, 8–19]. In our study multiple approaches were used to place fiducial markers. Of the five patients with percutaneous fiducial placement, we noted one grade I fiducial migration and one grade III bleeding complication requiring angiographic coil placement. Twelve patients had fiducial markers placed surgically without any complications. In our experience, surgical placement of fiducial markers is enhanced by use of the intraoperative ultrasound to detect tumor(s). 

RECIST is most commonly used method to report response based on measurement of maximal diameter in the abdominal tumor(s) [7]. However, this grading system is not the optimal way to assess the *entire* tumor's response to therapy. Measurement of largest two dimensional diameters is a single snapshot view into tumor effect that can occur after chemotherapy or locoregional therapy compared to a three-dimensional assessment of effect based on tumor volume. Gross tumor volume (GTV) is precisely measured during treatment planning for SBRT. The same process can be used after locoregional therapy including SBRT to assess tumor volume. We report the change in tumor volume after SBRT based on measuring GTV using the Multiplan system (Table 2). Amongst the current literature, there are vague and inconsistent definitions of tumor response and recurrence. Without a precise and uniform classification system it is difficult to compare results among studies and centers. We have developed a grading system for abdominal tumor response and recurrence using change in tumor *volume* after SBRT. We encourage its use in similar cohorts in an attempt to standardize comparisons among centers and among different treatment modalities.

It has been suggested that radiographic response analysis should be set no earlier than 4–6 months after SBRT to assess tumor response in clinical trials [18]. Future long-term data from randomized clinical trials are needed to determine the role of SBRT in the treatment of tumors of the liver. It appears that SBRT can play a primary role in the local control of these malignancies. The sample size and follow-up of this study are similar to other reported SBRT studies and add to the existing literature pertaining to SBRT for hepatobiliary malignancies. Within the limitations of a small sample size and short follow-up we have demonstrated that SBRT is a safe form of therapy for unresectable tumors of the liver with an 89% local response rate with six months mean follow-up. Future studies should focus on the development of strategies to define the role of SBRT in the treatment of liver tumors.

## 5. Conclusions

CyberKnife radiosurgery is a safe and effective local treatment option for unresectable primary and secondary liver tumors. In the multidisciplinary management of malignant maladies of the liver, SBRT adds to our armamentarium of local treatment modalities as complementary or salvage therapy. The role of SBRT as primary form of therapy remains to be determined. Further prospective studies are ongoing to determine long-term response and survival after SBRT for hepatobiliary malignancies.

## Figures and Tables

**Table 1:** Demographics of patients with nonresectable liver tumors treated with Stereotactic Body Radiation Therapy and baseline characteristics of the liver malignancies.

	HCC	IHC	LM
Subjects	6	3	8
Age (years)	*62.7 (52–81)	69.3 (61–86)	58.1 (42–77)
Gender (M : F)	4 : 2	2 : 1	3 : 5
Time from Dx to SBRT (months)	*5 (2–11)	8 (3–16)	5.4 (2–11)
Therapy prior SBRT			
Surgery	0	1	3
RFA	2	0	5
TACE	2	0	0
ChemoTx	3	2	8
Radiation	0	0	0
Tumor characteristics			
Number	*1 (1)	1 (N/A)	1 (1–6)
Diameter Max (cm)	*9.3 (5–22)		4.6 (2–9)
Volume (cc)	*386 (106–1268)	384 (80–818)	167 (26–433)

Abbreviations: HCC = Hepatocellular carcinoma; IHC = Intrahepatic cholangiocarcioma; LM = Liver metastases; M : F = Male : Female ratio; Dx = diagnosis; SBRT = Stereotactic Body Radiation Therapy; RFA = Radiofrequency ablation; TACE = Trans-arterial chemoembolization. *Mean (range).

**Table 2:** Tumor response, recurrence of malignancy, and adverse events in patients treated with Stereotactic Body Radiation Therapy for nonresectable liver tumors.

	HCC	IHC	LM	Total
Subjects	6	3	8	17
Mean of Follow Up (months)	10.0	5.9	6.9	7.8
Tumor Response				
RECIST	Complete (0)	Complete (0)	Complete (0)	Complete (0)
	Partial (5)	Partial (2)	Partial (7)	Partial (4)
	Stable (1)	Stable (0)	Stable (1)	Stable (2)
	Progress (0)	Progress (1)	Progress (0)	Progress (1)
Partial Tumor	Grade I (0)	Grade I (2)	Grade I (0)	Grade I (2)
Response Grade	Grade II (3)	Grade II (0)	Grade II (2)	Grade II (5)
	Grade III (3)	Grade III (1)	Grade III (6)	Grade III (10)
Tumor Recurrence				
	Grade 0 (4)	Grade 0 (1)	Grade 0 (4)	Grade 0 (9)
	Grade 1a (0)	Grade 1a (0)	Grade 1a (0)	Grade 1a (0)
	Grade 1b (0)	Grade 1b (1)	Grade 1b (0)	Grade 1b (1)
	Grade 2 (1)	Grade 2 (1)	Grade 2 (2)	Grade 2 (4)
	Grade 3 (1)	Grade 3 (0)	Grade 3 (0)	Grade 3 (1)
	Grade 4 (0)	Grade 4 (0)	Grade 4 (2)	Grade 4 (2)
Adverse events				
From SBRT	0	0	0	0
From markers	0	0	2	2
From surgery	0	0	0	0
# Patients Expired	2	3	3	8

## Abbreviations

SBRT: Stereotactic Body Radiotherapy,
UH-CMC: University Hospitals-Case Medical Center,
TACE: Trans-Arterial Chemoembolization,
RFA: Radiofrequency Ablation,
TEI: Transcutaneous Ethanol Injection,
CT: Computerized Tomography,
MRI: Magnetic Resonance Imaging,
PET: Positron Emission Tomography,
AFP: *α*-fetoprotein,
HCC: Hepatocellular Carcinoma,
IHC: Intra-hepatic Cholangiocarcinoma,
LM: Liver metastases,
Gy: Gray,
DVT: Deep Venous Thrombosis,
GI: Gastrointestinal,
HPB: Hepatopancreatobiliary.
